# Modulation of the mRNA-binding protein HuR as a novel reversal mechanism of epirubicin-triggered multidrug resistance in colorectal cancer cells

**DOI:** 10.1371/journal.pone.0185625

**Published:** 2017-10-02

**Authors:** Guan-Liang Lin, Huei-Ju Ting, Ta-Chien Tseng, Vivian Juang, Yu-Li Lo

**Affiliations:** 1 Department of Biological Sciences and Technology, National University of Tainan, Tainan, Taiwan; 2 Institute of Bioinformatics and Biosignaling Transduction, National Cheng Kung University, Tainan, Taiwan; 3 Department and Institute of Pharmacology, National Yang-Ming University, Taipei, Taiwan; University of South Alabama Mitchell Cancer Institute, UNITED STATES

## Abstract

HuR (ELAVL1), a RNA-binding protein, plays a key role in posttranscriptional regulation of multidrug resistance (MDR)-related genes. Among various HuR-regulated oncogenic transcripts, the activation of galectin-3/β-catenin survival pathway is critical to induce transcription of cyclin D1, P-glycoprotein (P-gp) and/or multidrug resistance-associated proteins (MRPs). In this study, we aim to elucidate the HuR-regulating pathways related to epirubicin-mediated resistance in human colorectal carcinoma cells. The effects and mechanisms of epirubicin treatment on the expressions of upstream survival signals (e.g., β-catenin) and downstream MDR transporters (e.g., P-gp) and anti-apoptotic pathways (e.g., Bcl-2) were assessed with or without HuR knockdown (siHuR) or overexpression (overHuR; ectopic HuR or pcDNA3/HA-HuR). Our results showed that siHuR decreased transcriptional expressions of galectin-3, β-catenin, cyclin D1, Bcl-2, P-gp, MRP1, and MRP2 in epirubicin-treated colon cancer cells. Consistently, the co-treatment of epirubicin and siHuR diminished the expressions of galectin-3, ß-catenin, c-Myc, P-gp and MRP1. HuR silencing enhanced the intracellular accumulation of epirubicin in colon cancer cells. On the other hand, overHuR abolished such effects. Furthermore, siHuR significantly intensified epirubicin-mediated apoptosis via increasing reactive oxygen species and thus promoted the cytotoxic effect of epirubicin. The combined treatments of siHuR and epirubicin significantly reduced the expression of Bcl-2, but increased the expression of Bax, as well as activity and expression levels of caspase-3 and -9. In contrast, overHuR abrogated these effects. Our findings provide insight into the mechanisms by which siHuR potentiated epirubicin-induced cytotoxicity via inhibiting galectin-3/β-catenin signaling, suppressing MDR transporters and provoking apoptosis. To our best knowledge, this is an innovative investigation linking the post-transcriptional control by HuR silencing to survival signaling repression, efflux transporter reversal and apoptosis induction. Our study thus provides a powerful regimen for circumventing MDR in colon cancer cells.

## Introduction

The mRNA-binding protein HuR (human antigen R, *ELAVL1*) acts by stabilizing AU-rich elements (AREs) in the 3' untranslated regions (3'-UTR) of mRNA [1,2] and thus mediates post-transcriptional upregulation of key survival or growth-related genes by increasing both mRNA stability and/or protein translation [3,4]. Accordingly, HuR regulates cellular functions of tumor progression, apoptosis, invasion, and metastasis [5].

The development of multidrug resistance (MDR) to conventional chemotherapy causes treatment failure in various cancers. Overexpression of ATP-binding cassette (ABC) efflux proteins including P-glycoprotein (P-gp, encoded by the *MDR1* (ABCB1) gene) and multidrug-resistance associated proteins (MRPs) work by active transport of anticancer drugs out of cells and thus decrease efficacy of these drugs [6]. Numerous studies have indicated that cytoplasmic accumulation of HuR has a link to MDR of cancer cells acquired after chemotherapy and thus causes poor prognosis of survival in various cancers [7–9]. Accordingly, suppression of the cytoplasmic accumulation of HuR during the treatment of antineoplastic therapeutics may be a potential approach for reversing drug resistance [7,10]. Furthermore, upregulation of cytoplasmic HuR and overexpression of P-gp were found in patients with breast and ovarian cancer [7,11]. Consistently, therapy using siRNA against HuR suppressed ovarian tumor growth [11]. Moreover, HuR acts by binding to the 3'-UTR of many Bcl-2 family members and HuR silencing causes unstable transcript of Bcl-2 and inhibits Bcl-2 protein expression, thus triggering apoptosis and inhibiting brain glioma cell growth [12].

HuR has been advocated to regulate mRNA stabilization of oncogenic transcripts, including β-catenin, cyclin D1, and c-Myc, which are crucial in Wnt-activated pathway in colon cancer cells [4,13,14]. Furthermore, β-catenin mRNA has been identified as a HuR target and siRNA against HuR reduced colon cancer growth [4,15]. Moreover, β-catenin stabilized mRNA of c-Jun and cyclin D1, as mediated by HuR [16]. Additionally, accumulating evidences have verified a positive correlation between the expressions of β-catenin, c-Myc, and cyclin D1 and the upregulation of P-gp [17–19]. Our previous investigation has demonstrated for the first time that siRNA against galectin-3 modulated GSK-3β phosphorylation and suppressed β-catenin expression, thus inhibiting epirubicin-triggered resistance via decreasing the expressions of cyclin D1, Bcl-2, c-Myc, P-gp, MRP1, and MRP2 in human colon cancer cells [17]. Accordingly, it is important to further clarify the role of HuR in affecting signaling pathway of galectin-3, GSK-3β, and/or β-catenin and the downstream MDR-related gene expressions.

In the present study, we proposed HuR silencing (siHuR) or HuR overexpression (overHuR) as regulators of MDR pump resistance and anti-apoptosis non-pump resistance. The model anticancer drug, epirubicin (Pharmorubicin®; abbreviated as Epi) is an epimer of doxorubicin and is a substrate of P-gp, MRP1, and MRP2 [20,21]. Epi displayed a powerful apoptotic effect against various tumor cells via the intrinsic mitochondrial signaling pathway accompanying with galectin-3-mediated Wnt/β-catenin pathway modulation [17,21,22]. In this study, we aim to elucidate the HuR-associated signaling pathways related to chemoresistance of human colorectal carcinoma cells to Epi. The expressions of upstream survival signals (GSK-3β, β-catenin, c-Myc and cyclin D1), downstream ABC transporters, including P-gp and MRPs, and apoptosis-related proteins, such as Bcl-2, Bax, and caspases in colon cancer cells were assessed after Epi treatment in the presence and absence of HuR knockdown or overexpression. We set sights on increasing the chemosensitivity of colon cancer cells to Epi through suppression of MDR transporters and apoptosis induction via inhibition of HuR-mediated galectin-3/β-catenin pathway.

## Materials and methods

### Reagents

Synthetic small interfering RNA targeting the HuR mRNA (siHuR) was purchased from Thermo Fisher Scientific Inc. (Carlsbad, CA, USA). The HuR expression plasmid, pcDNA3/HA-HuR, was provided by Dr. Ta-Chien Tseng at the Institute of Bioinformatics and Biosignal Transduction, National Cheng Kung University (Tainan, Taiwan, ROC). The Genmute™ and Polyjet™ siRNA transfection reagents were obtained from SignaGen Laboratories (Rockville, MD, USA). All cell culture medium and reagents were bought from Gibco (Grand Island, NY, USA) or Hyclone (Logan, UT, USA). The chemical reagents including 3-(4,5-dimethylthiazol-2-yl)-2,5-diphenyl tetrazolium bromide (MTT), dimethyl sulfoxide (DMSO), ethanol and ribonuclease A (RNase A) were purchased from Sigma-Aldrich (St. Louis, MO, USA). 3,3'-dihexyloxacarbocyanine iodide (DiOC6), 2',7'-dichlorodihydrofluorescein diacetate (DCFH-DA) and dihydroethidium (HE) were bought from Invitrogen (Carlsbad, CA, USA).

### Cell lines

The colorectal carcinoma SW620 and HCT116 cell lines were provided by Dr. Ta-Chien Tseng and Prof. Yu Su, National Yang-Ming University, respectively. Cells were cultured in Leibovitz's L-15 Medium supplemented with 10% fetal bovine serum (FBS; Hyclone), and penicillin/streptomycin (Gibco) at 37°C in a humidified atmosphere of 5% CO2 and 95% air.

### Transfection

Cells were plated at 6×10^5^ cells/well in six-well plates on the day before transfection. For HuR knockdown study, cells were transfected with scramble siRNA (siC), or 0, 25, 50, 75 nM siHuR using Genmute™ siRNA transfection reagent according to the manufacturer’s manual. The sequence of siC does not down- or upregulate any known mammalian gene. For HuR overexpression study, cells were transfected with pCDNA/HA or 0, 1, 2, 3, 4 μg pCDNA/HA-HuR using Polyjet™ transfection reagent according to the manufacturer’s instruction. For evaluation of the effect of HuR silencing on the protein expression levels of survival signaling-, efflux transporter- and apoptosis-related factors, including galectin-3, c-Myc, P-gp, MRP1, Bcl-2, caspase-3, and -9, colon cancer cells were transfected with 75 nM siC or siHuR for 24 h and then treated with Epi (5 μg/ml) for 24 h.

### Western blotting

Cells were plated at 6×10^5^ cells/well in six-well plates and transfected as described above.

The proteins were extracted, and the total protein concentration was determined by Bio-Rad protein assay reagent (Bio-Rad, Hercules, CA, USA) following manufacturer’s manual. The absorbance was measured by a spectrophotometer (CT-5600; ChromTech, Inc, Apple Valley, MN, USA). Proteins were separated in 8%–13.5% polyacrylamide-SDS gel and transferred onto a polyvinylidene difluoride (PVDF) membrane. After blocking with 5% skim milk in TBST (50 mM Tris, pH 7.4, 150 mM NaCl, 0.1% Tween 20), the membranes were incubated with specific antibodies against Calnexin, HuR, HA, c-Myc, caspase-3, -9 (Cell Signaling Technology Inc., Beverly, MA, USA), galectin-3, P-gp, MRP1, Bcl-2 (GeneTex Inc., Hsinchu, Taiwan), and β-actin (Millipore, Billerica, MA, USA) overnight at 4°C. These membranes were then incubated with horseradish peroxidase (HRP)-conjugated goat anti-rabbit IgG (Jackson ImmunoResearch Inc., PA, USA) for 1 h at room temperature. The detection of the signal was performed by incubating blotted membranes with enhanced chemiluminescence detection kit (PerkinElmer Life Sciences, Waltham, MA, USA).

### Cytotoxicity assay

The cell viability was evaluated by MTT assay. Cells were seeded at 2×10^4^/well in 96-well plates. After transfection of the siHuR (25, 50 and 75 nM) or HuR (1, 2, 3 and 4 μg) for 24 h, cells were treated with increasing concentrations of Epi (0, 1, 2.5, 5 and 10 μg/ml) for another 24 h. The cells were incubated with 0.2 mg/ml MTT and maintained in 5% CO_2_ incubator at 37°C for 4 h. DMSO (100 μl) was added to each well to solubilize the formazan, and the absorbance was measured at 540 nm by Epoch™ Microplate Spectrophotometer (BioTek, Winooski, VT, USA). The relative cell viability (%) was calculated by setting the control as 100%. Data were expressed as the means ± standard deviation (S.D.) of six experiments.

### Analysis of cellular reactive oxygen species (ROS)

Cells were plated at 6×10^5^ cells/well in six-well plates. After transfection of 75 nM siHuR or 2 μg HuR for 24 h, cells were treated with 5 μg/ml Epi for 24 h. The cells were incubated with 0.1 μM DCFH-DA or 0.25 μM HE and maintained in 5% CO_2_ incubator at 37°C for an additional 30 min. Cells were harvested and analyzed using a flow cytometer (Cell Lab Quanta SC MPL; abbreviated as Quanta SC; Beckman Coulter, Fullerton, CA, USA).

### Intracellular Epi accumulation

After transfection and treatment as described above, cells were harvested in PBS. The fluorescence intensity representing intracellular amount of Epi (excitation: 474 nm; emission: 545 nm, FL-2) was measured using a flow cytometer (Quanta SC). Data acquisition and analysis were performed using commercial software (Quanta SC). At least 10,000 cells were determined in each sample and the results are obtained from three independent experiments.

### Reverse transcription and quantitative real-time PCR

Cells were plated at 6×10^5^ cells/well in six-well plates. After transfection and treatment, total RNA was extracted from cells using the Total RNA Extraction Miniprep System (Viogene, Taipei, Taiwan) according to the manufacturer’s instruction. The RNA yield and purity were assessed using Epoch™ Microplate Spectrophotometer (BioTek). The synthesis of cDNA was prepared from total RNA using the High-capacity RNA-to-cDNA kit (Applied Biosystems; Foster City, CA, USA) following the manufacturer's protocol. The relative mRNA expression level was measured by quantitative PCR using gene-specific primers (Table 1) for 18S, MDR1, MRP1, MRP2, Bax, Bcl-2, c-Myc, Cyclin D1, ß-catenin, GSK-3ß, and galectin-3. Quantitative PCR was conducted using the StepOne Real-Time PCR system (Applied Biosystems) and SYBR Green PCR Master Mix (Applied Biosystems). We predicted cycle threshold (CT) values after exporting the data directly into EXCEL worksheets for analysis. After cycling, a melting curve composed of slow denaturation of the PCR product was used to validate the specificity of amplification. The cycling program was finally determined as follows: denaturation at 95°C for 10 min followed by 40 cycles of 95°C for 15 s and 60°C for 1 min. The results are detected from three independent experiments in which each sample was assayed in triplicate and normalized by the 18S rRNA level. The relative expression levels of genes were calculated by setting the control group as one.

**Table 1:** The primer sequences used for quantitative real-time PCR.

Gene name	Forward primer	Reverse primer
18S	GCGCAAATTACCCACTCCCG	CCCGCTCCCAAGATCCAACT
Galectin-3	TAATAACTGGGGAAGGGAAG	AGCACTGGTGAGGTCTATGT
GSK-3ß	ACTTTGTGACTCAGGAGAACTGG	TCGCCACTCGAGTAGAAGAAATA
ß-catenin	TCAAATTTTAGCTTATGGCAA	TTATTACTAGAGCAGACAGAT
Cyclin D1	CACACGGACTACAGGGGAGT	CACAGGAGCTGGTGTTCCAT
C-myc	TCAAGAGGCGAACACACAAC	GGCCTTTTCATTGTTTTCCA
MDR1^a^	GCTCATCGTTTGTCTACAGTTCGT	ACAATGACTCCATCATCGAAACC
MRP1^b^	GGATCATGCTCACTTTCTGG	AAGTGATGTCACGAAACAGGTC
MRP2^c^	AAGATGCAGCCTCCATAACCA	TGGACCTAGAACTGCGGCTAA
Bcl-2	CTTGACAGAGGATCATGCTGTAC	GGATGCTTTATTTCATGAGGC
Bax	GGGCCCACCAGCTCTGA	CCTGCTCGATCCTGGATGA

^a^MDR1, multidrug resistance gene 1.

^b^MRP1, gene of MDR-associated protein 1.

^c^MRP2, gene of MDR-associated protein 2.

### Cell cycle analysis by flow cytometry

After transfection and treatment as designed, the cells were harvested by centrifugation and gently fixed with 70% ethanol at -20°C overnight. After fixation, the cells were treated with 1 ml hypotonic buffer (0.5% Triton X-100 and 100 μg/ml RNase A in PBS) and stained with 50 μg/ml propidium iodide in PBS for 30 min at 25°C in the dark. The cells were analyzed using a flow cytometer (Quanta SC). The presented results are from three individual experiments.

### Apoptosis detection assay

The Annexin V-FITC Apoptosis Detection Kit (Biovision, Mountain View, CA, USA) was used to analyze the percentage of cells in different stages. After transfection and treatment as designed, cells were harvested and stained with Annexin V-propidium iodide (PI) labeling solution for 15 min at room temperature in the dark. Different cell populations, including intact cells (FITC^-^/PI^-^), early apoptotic cells (FITC^+^/PI^-^), late apoptotic or necroptotic cells (FITC^+^/PI^+^) and late apoptotic or necrotic cells (FITC^-^/PI^+^) were detected by flow cytometer (Quanta SC) [23,24]. Data acquisition and analysis were performed using commercial software (Quanta SC). Phosphatidylserine was exposed in early apoptotic cells with intact cell membranes and was bound to Annexin V-FITC. But these cells excluded PI (Annexin V-FITC positive, PI negative). Cells in late apoptotic stages are both Annexin V-FITC and PI positive.

### Caspase-3/7, caspase-8, and caspase-9 activity assay

Caspase-3/7, caspase-8, and caspase-9 activities were detected by the luminescence-based Caspase-Glo® 3/7, Caspase-Glo® 8 and Caspase-Glo® 9 Assay Kits (Promega), respectively. After transfection and treatment as designed, cells were harvested and resuspended in L-15 medium. After lysis, 20 μl cell suspension was then mixed with 20 μl of the caspase-3, caspase-8 or caspase-9 reagents containing the corresponding luminogenic substrates, Ac-DEVD-pNA, Ac- LETD-pNA, and Ac-LEHD-pNA at room temperature for 30 min. Levels of released aminoluciferin luminescence were measured using a microplate luminometer (CentroPRO LB 962; Berthold Technologies GmbH & Co. KG, Bad Wildbad, Germany).

### Measurement of mitochondrial membrane potential (MMP)

Cells were plated at 6×10^5^ cells/well in six-well plates. After transfection and treatment as designed, the cells were incubated with 0.2 μM DiOC_6_ (one anionic lipophilic dye) and maintained in 5% CO_2_ incubator at 37°C for an additional 30 min. Cells were harvested and MMP represented by the relative fluorescence intensity % of DiOC_6_ was detected using a flow cytometer (Quanta SC). The presented results are from three individual experiments.

### Statistical analysis

Data are shown as the means ± standard deviation (S.D.). Student’s t-test was used to analyze differences between two treatment groups. Significant difference was set at P < 0.05.

## Results

### The expression levels of HuR slightly affected viability of colon cancer cells

**Fig 1:**
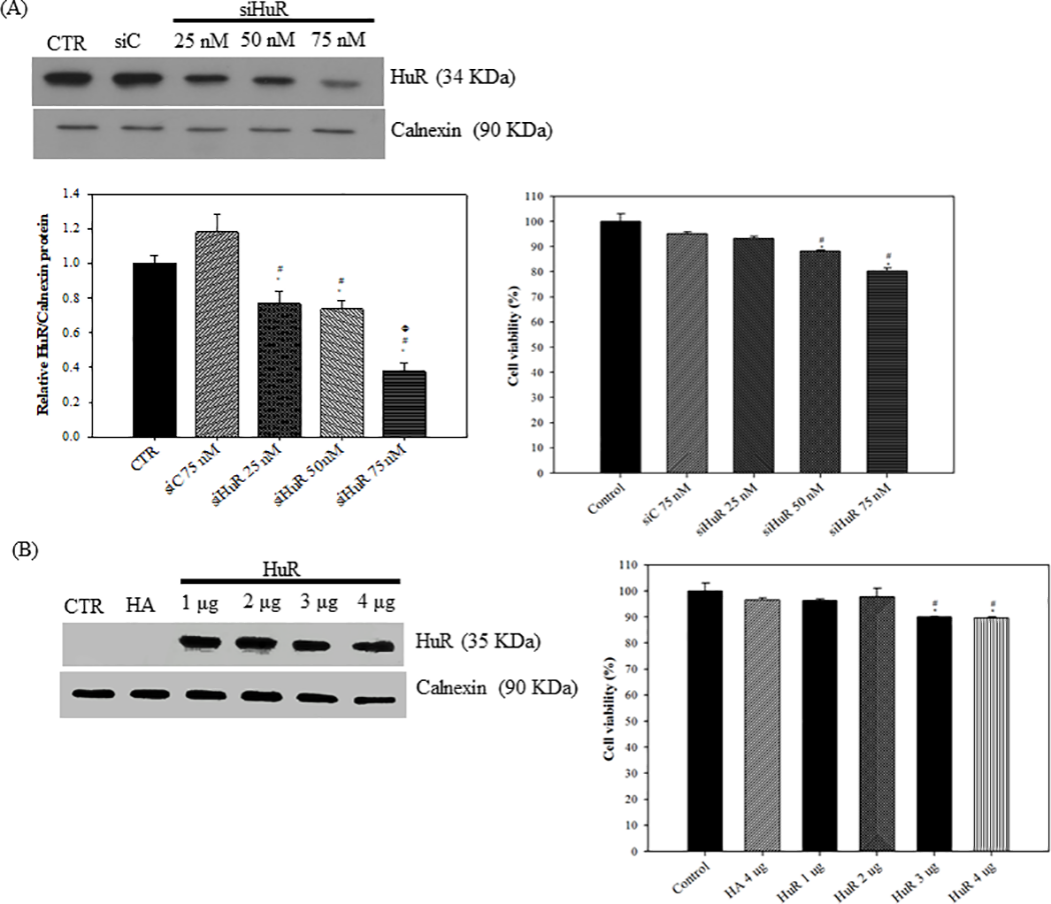
The effect of HuR expression levels on viability of SW620 cells. **(A) The knockdown efficiency of siHuR and its effect on cell viability.** SW620 cells were transfected with 75 nM of scramble siRNA (siC) or 0, 25, 50, 75 nM of siRNA against HuR (siHuR) for 48 h. Cells without transfection are designed as control (CTR). Proteins were then extracted for detection of HuR and Calnexin by western blotting (upper panel) and the quantification of relative HuR/Calnexin expressions was shown. Data are presented as means ± S.D. from three independent experiments (lower left panel). * P<0.05 compared to the control group; ^#^ P<0.05 compared to the siC group; ^Φ^ P<0.05 compared to siHuR 25 nM group. The effect of siHuR on the viability of SW620 cells were measured at 0, 25, 50, 75 nM of siHuR for 24 h. Viable cells were measured by MTT assay. The percentage of cell viability was calculated by setting the control group as 100%. Data are presented as means ± S.D. from three independent experiments (lower right panel). * P<0.05 compared to the control group; ^#^ P<0.05 compared to the siC group. **(B) The overexpression efficiency of HuR and its effect on cell viability.** Cells were transfected with pCDNA-HA (HA) 4 μg or 0, 1, 2, 3, 4 μg/ml of pCDNA-HA-HuR (HuR) for 48 h. Cells without transfection were designed as control. Proteins were then extracted for detection of HuR and Calnexin by western blotting (upper panel). The effect of HuR on the cell viability was measured at 0, 1, 2, 3, 4 μg/ml of HuR for 24 h. The viable cells were measured and analyzed as in (A). The plot was shown in the lower panel of (B). * P<0.05 compared to the control group; ^#^ P<0.05 compared to the HA group.

The protective effect of HuR against cellular stresses including oxidative stress, cytotoxic agents, and apoptotic signaling has been documented [3,25]. Here, we studied the effect of HuR on colon cancer cell viability by knockdown or overexpression of HuR in SW620 cells. First, the endogenous amount of HuR was decreased by using siRNA targeting HuR (siHuR). Among different concentrations of siHuR transfected, the knockdown efficiency was most significant when using siHuR at 75 nM (Fig 1A, upper panel; lower left panel). The cell viability decreased about 20% when cells were transfected with siHuR at 75 nM (Fig 1A, lower right panel) compared to control group. Next, the cells were treated by HuR overexpression (overHuR) tagged with HA (Fig 1B, upper panel). The overexpression of HuR also slightly decreased cell viability about 10% when cells were transfected with 3 or 4 μg of overHuR plasmids compared to either control or HA group (Fig 1B, lower chart). We thus determined to use 2 μg of overHuR plasmid for the following studies.

### HuR silencing intensified the cytotoxic effect of Epi on colon cancer cells

**Fig 2:**
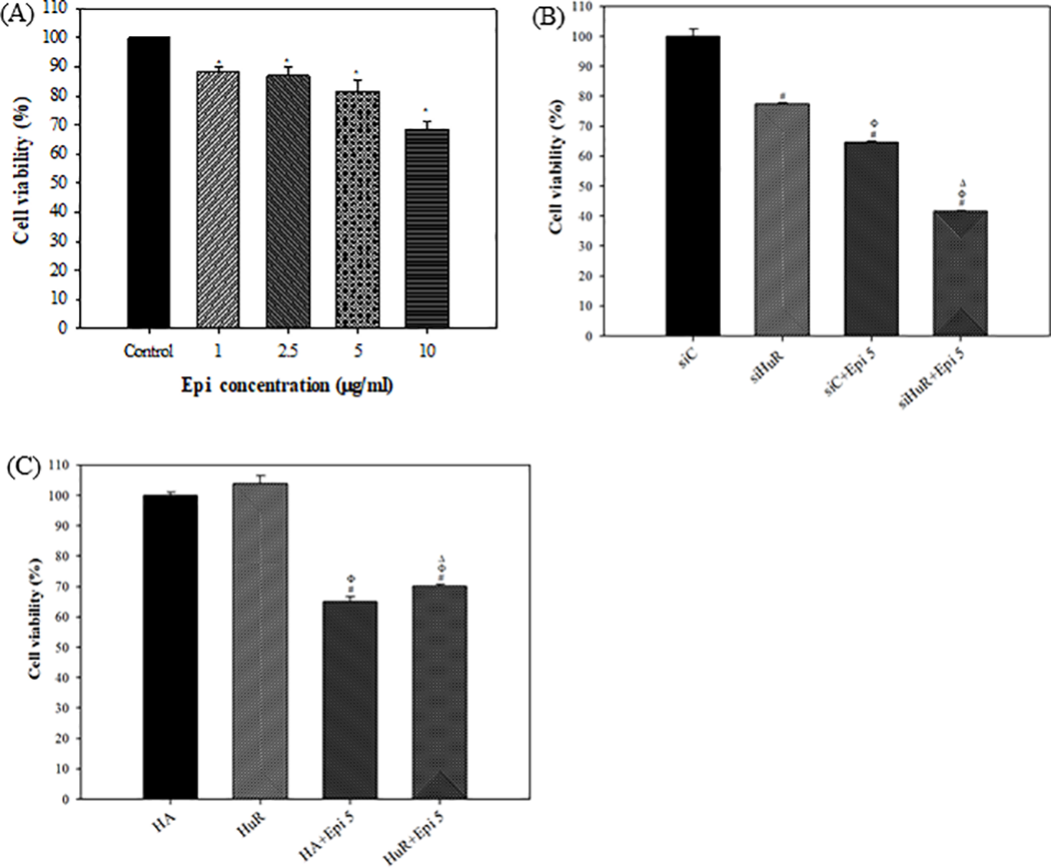
HuR protected SW620 cells from the cytotoxic effect of Epi. **(A) The dose-dependent cytotoxic effect of Epi on SW620 cells.** Cells were treated with different concentrations of Epi as indicated for 24 h. Cells without treatment were designed as Control. Viable cells were measured by MTT assay. The percentage of cell viability was calculated by setting the Control group as 100%. Data are presented as means ± S.D. from three independent experiments. Each experiment was conducted in triplicate. * P<0.05 compared to the Control group. **(B-C) The expression levels of HuR affected the cytotoxic effect of Epi on SW620 cells.** Cells were transfected with 75 nM siC or siHuR (B), or with 2 μg/ml HA and HuR (C). After 24 h, cells were treated with Epi (5 μg/ml) for 24 h. Viable cell number was measured by MTT assay. The percentage of cell viability was calculated by setting the siC group (B) or HA group (C) as 100%. Data are presented as means ± S.D. from three independent experiments. Each experiment was conducted in triplicate. **(B) HuR knockdown enhanced the cytotoxic effect of Epi.** ^#^ P<0.05 compared to the siC group; ^Φ^ P<0.05 compared to the siHuR group; ^Δ^ P<0.05 compared to the siC + Epi group. **(C) HuR overexpression protected cells from the cytotoxic effect of Epi.** ^#^ P<0.05 compared to the HA group; ^Φ^ P<0.05 compared to the HuR group; ^Δ^ P<0.05 compared to the HA + Epi group. HA: pCDNA/HA; HuR: pCDNA/HA-HuR.

One bottleneck in battling cancer is that cancer cells can acquire resistance to cytotoxic effect of chemotherapeutic drugs. Based on the fact that HuR is involved in the development of drug resistance in cancer cells [3,26], we investigated the impact of modulating HuR expression levels on the cytotoxic effect of Epi, one anticancer drug. Here, we first examined the dose-dependent cytotoxic effect of Epi on SW620 cells. The cell viability after treatment with 5 μg/ml of Epi was 81.45 ± 4.04%, but reduced to 68.44 ± 2.93% at 10 μg/ml for 24 h treatment (Fig 2A). We thereafter chose 5 μg/ml Epi to evaluate the effect of silencing or overexpressing HuR on the response of SW620 cells to Epi treatment. When HuR expression was inhibited by siHuR at 75 nM, Epi treatment reduced SW620 cell viability by 58.44 ± 0.40% (Fig 2B, lane 4). This demonstrated that the cytotoxic effect of Epi can be synergistically increased by HuR knockdown in SW620 cells. We also found that HuR downregulation by siHuR potentiated Epi’s cytotoxicity in HCT116 cells (data not shown). On the other hand, overexpression of HuR slightly protected cells from the cytotoxicity of Epi (Fig 2C, lane 4).

### Generation of ROS was induced by HuR knockdown and potentiated by Epi treatment

**Fig 3:**
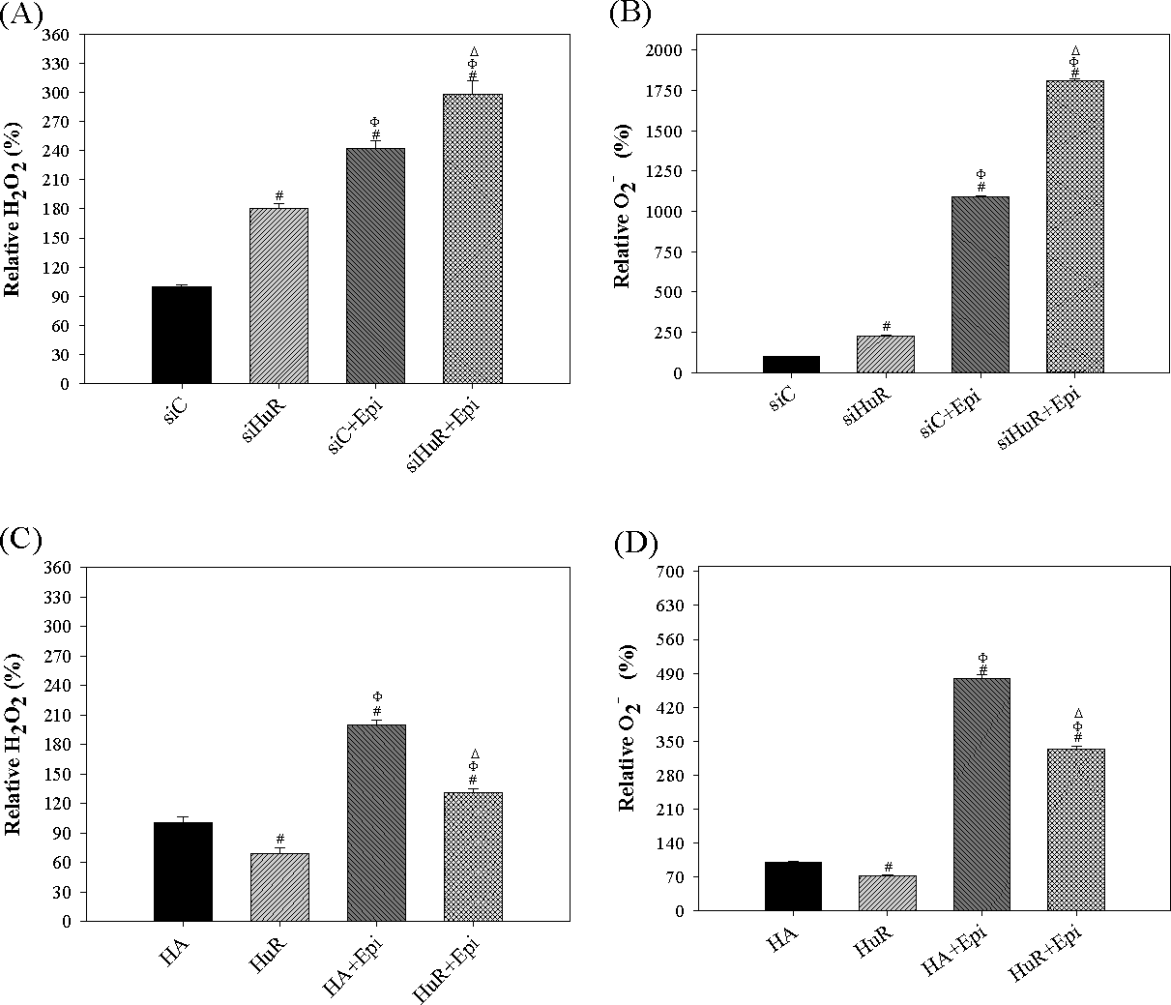
The effect of HuR expression levels on Epi-induced ROS production in SW620 cells. **(A-B) HuR knockdown increased Epi-induced ROS production in SW620 cells.** Cells were transfected with 75 nM siC or siHuR. Cells were then treated with Epi (5 μg/ml) for 24 h. (A) The cellular H_2_O_2_ level was detected by DCF intensity and analyzed by Flow Cytometry. (B) The cellular O_2_^-^ level was measured by EtBr intensity and analyzed by Flow Cytometry. The relative ROS percentage was calculated by setting the mean fluorescence intensity of siC group as 100%. Data are presented as means ± S.D. from three independent experiments. ^#^ P<0.05 compared to the siC group; ^Φ^ P<0.05 compared to the siHuR group; ^Δ^ P<0.05 compared to the siC + Epi group. **(C-D) HuR overexpression decreased Epi-induced ROS production in SW620 cells.** Cells were transfected with 2 μg/ml HA or HuR. After 24 h, cells were treated with Epi (5 μg/ml) for 24 h. (C) The cellular H_2_O_2_ level was detected by DCF intensity and analyzed by Flow Cytometry. (D) The cellular O_2_^-^ level was measured by EtBr intensity and analyzed by Flow Cytometry. The relative percentage of ROS was calculated by setting the mean fluorescence intensity of HA group as 100%. Data are presented as means ± S.D. from three independent experiments. ^#^ P<0.05 compared to the HA group; ^Φ^ P<0.05 compared to the HuR group; ^Δ^ P<0.05 compared to the HA + Epi group.

The cytotoxic effect of Epi was partly mediated by the generation of ROS that subsequently damage mitochondria leading to apoptosis [21,27]. It has also been reported that ROS generation triggered cytoplasmic translocation of HuR, leading to enhancement in activity of TGFβ pathway (ROS-HuR-TGFβ pathway) [28]. Moreover, HuR was implicated in posttranscriptional regulation of NADPH oxidase-1 (NOX-1), one crucial enzyme for ROS production [29]. We therefore suspected that modulating HuR might affect the generation of ROS induced by Epi. To evaluate intracellular ROS levels, a flow cytometric assay using cell permeable probes DCFH-DA and DHE were developed to measure the H_2_O_2_ and O_2_^−^ production, separately. We measured fluorescent dichlorofluorescein (DCF) and ethidium bromide (EtBr) products after ROS conversion inside the cells. Here, we demonstrated that HuR silencing increased cellular ROS levels, including H_2_O_2_ and superoxide (O_2_^-^) (Fig 3A and 3B, lane 2). On the other hand, HuR overexpression decreased ROS generation (Fig 3C and 3D, lane 2). Furthermore, Epi induced ROS generation, which was enhanced by siHuR (Fig 3A and 3B, lane 3 vs lane 4) and was suppressed by HuR overexpression (Fig 3C and 3D, lane 3 vs lane 4). Therefore, the combined treatment of siHuR and Epi indeed cooperatively induced the ROS production in SW620 cells.

### Cellular uptake of Epi was influenced by HuR through modulation of MDR1 and MRP expressions

**Fig 4:**
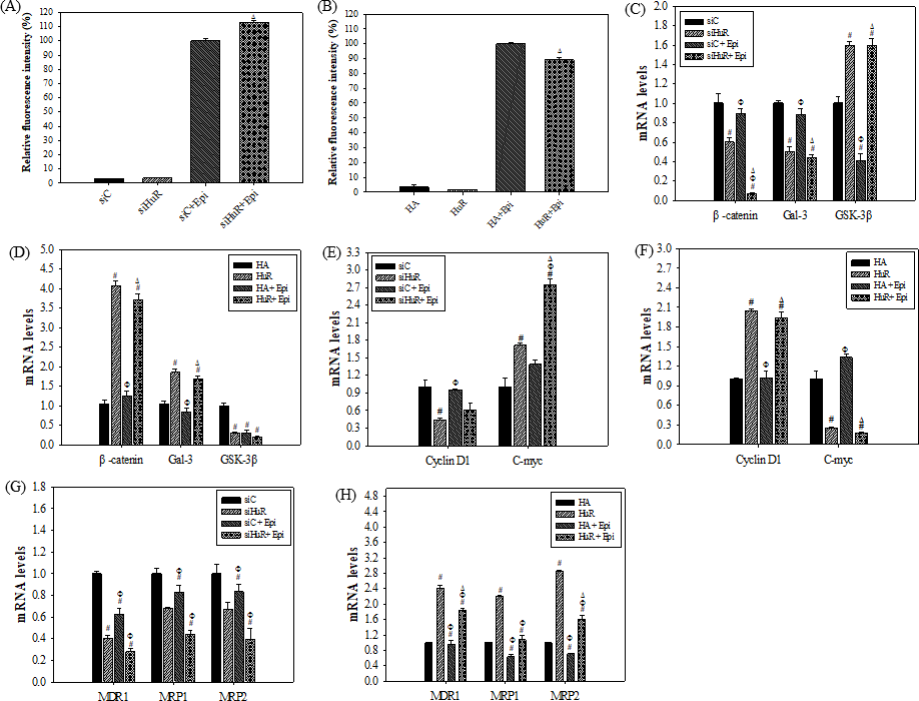
HuR modulated the intracellular Epi accumulation by regulating the expressions of genes associated with β-catenin/GSK-3β pathway, oncogenes, and MDR transporters in SW620 cells. **(A-B) HuR silencing or overexpression affected the intracellular accumulation of Epi in SW620 cells.** SW620 cells were transfected with 75 nM siC or siHuR (A) or with 2μg HA or HuR (B). After 24 h, cells were treated with Epi (5 μg/ml) for 24 h. The relative fluorescence intensity of each group in the cells was measured by flow cytometry. It was calculated by setting the fluorescence intensity of siC + Epi (A) or HA + Epi (B) group as 100%. Data are presented as mean percentages ± S.D. from three independent experiments. ^**Δ**^P<0.05 compared to the siC + Epi (A) or HA + Epi (B) group. The relative fluorescence intensity % of siC, siHuR, HA, and HuR were also shown here to demonstrate that the auto-fluorescence of siC, siHuR, HA, and HuR was negligible. **(C-D) HuR knockdown or overexpression affected the mRNA expressions of β-catenin/GSK-3β pathway-related genes, including galectin-3, GSK-3β, and β-catenin in SW620 cells. (E-F) HuR knockdown or overexpression affected the mRNA expressions of oncogenes, such as cyclin D1 and c-Myc in SW620 cells. (G-H) HuR silencing or overexpression affects the mRNA expressions of MDR transporter-related genes in SW620 cells.** For (C-H), the cells were transfected with 75 nM siC or siHuR (C, E, G), or 2 μg HA or HuR (D, F, H), separately. After 24 h, the cells were treated with Epi (5 μg/ml) for 24 h. RNA was extracted from the cells as treated above for measurement of mRNA expressions by real-time PCR. The relative expression levels of genes were calculated by setting siC (C, E, G) or HA (D, F, H) group as 1, respectively. Means ± S.D. from three independent experiments were plotted. ^#^P<0.05 compared to the siC or HA group; ^Φ^P<0.05 compared to the siHuR or HuR group; ^Δ^P<0.05.

Accumulating evidences have supported that HuR participates in drug resistance of various cancer via multiple mechanisms [7,8,12]. Here, we have found that down- or up-regulation of HuR affected cytotoxic effect and ROS inductive effect of Epi. Therefore, dissecting the mechanisms in which HuR protected SW620 cells from cytotoxic effect of Epi could lead to novel therapeutic strategy combating drug resistance. Thereafter, we investigated if HuR could decrease cellular accumulation of Epi and hence protected cells from the cytotoxic effect of Epi. Interestingly, when the cells were transfected with siHuR, the intracellular accumulation of Epi was increased to 112.92 ± 1.29% (Fig 4A), while overHuR reduced the uptake of Epi to 89.11 ± 1.68% (Fig 4B). HuR reduced intracellular accumulation of Epi while siHuR increased it (Fig 4A and 4B). It has been reported that ABC transporters, including MDR1, MRP1 and MRP2, facilitate export of Epi [21]. We thus suspect that HuR influenced the expressions of MDR transporters and/or their upstream signaling regulators. The mRNA expression levels of galectin-3, ß-catenin, cyclin D1, c-Myc, MDR1, MRP1, and MRP2 upon treatment with Epi in cells of siHuR or overHuR were measured. We found that Epi or siHuR treatment for 24 h decreased the cellular mRNA levels of galectin-3 and ß-catenin (Fig 4C). The combined treatment of Epi and siHuR further diminished the mRNA levels of galectin-3 and ß-catenin (Fig 4C). On the contrary, overHuR increased the mRNA levels of galectin-3 and ß-catenin (Fig 4D). The co-addition of Epi and overHuR demonstrated an incremental effect on galectin-3 and ß-catenin expressions (Fig 4D). Moreover, siHuR treatment reduced the mRNA expression of cyclin D1, while overHuR treatment increased it (Fig 4E and 4F). Unexpectedly, Epi treatment did not significantly change the expression of cyclin D1 compared to control (SiC or HA, respectively; Fig 4E and 4F). Consistently, the inhibitory effect of Epi and siHuR or inductive effect of Epi and overHuR on cyclin D1 was similar to the degree of siHuR or overHuR alone, indicating the dominant role of HuR in cyclin D1 modulation (Fig 4E and 4F). To our surprise, siHuR or Epi treatment increased the mRNA expression of c-Myc, while overHuR treatment reduced the level (Fig 4E and 4F). The combined treatment of siHuR and Epi remarkably potentiated the enhancement on c-Myc expression (Fig 4E). Steadfastly, we found that HuR knockdown by siHuR or Epi treatment decreased cellular mRNA levels of MDR1, MRP1 and MRP2 compared to siC group (Fig 4G). Co-treatment of siHuR and Epi showed additional reduction on the mRNA expressions of these MDR transporters in SW620 cells. Interestingly, co-treatment of siHuR and Epi also showed significant reduction on the mRNA expressions of these MDR transporters compared to siC + Epi group in HCT116 cells (data not shown), implying the partial reversal of Epi-triggered resistance in both SW620 cells and HCT116 cells. On the other hand, overHuR increased the mRNA level of ABC transporters compared to both HA group and Epi treated group (Fig 4H). The induction of MDR transporter expressions by overHuR was partially reversed by the addition of Epi (Fig 4H). Overall, the expressions of ABC transporters were decreased by siHuR while increased by overHuR, but reduced by the Epi treatment. In summary, HuR increased the expressions of MDR transporters and decreased the cytotoxic effect of Epi by increasing export of Epi, thus reducing intracellular accumulation of Epi. Furthermore, silencing of HuR decreased the expressions of MDR1 and MRPs, hence increasing the cytotoxicity of Epi by trapping Epi inside the colon cancer cells.

### HuR knockdown enhanced Epi-induced apoptosis

**Fig 5:**
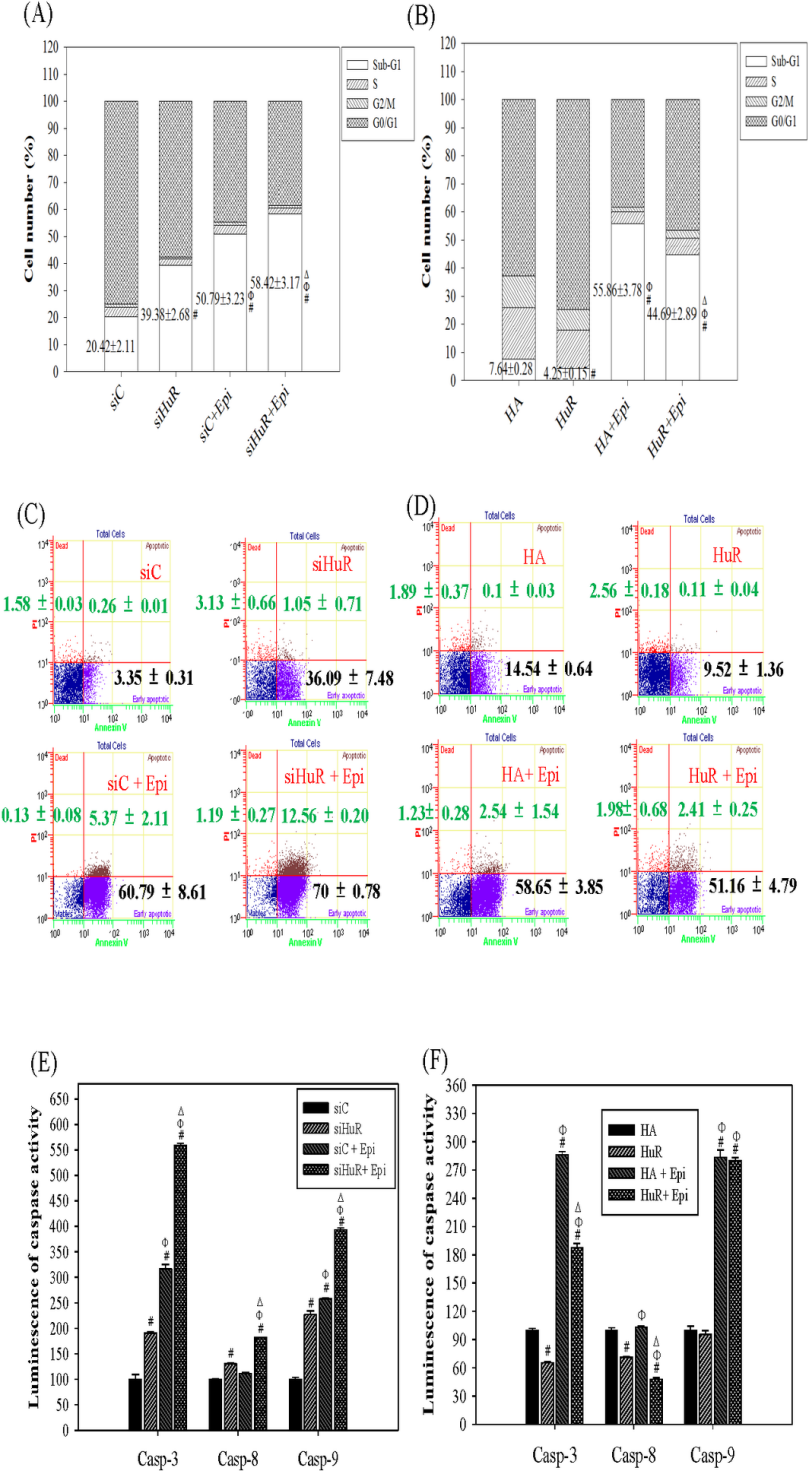
The effect of HuR knockdown and overexpression on the cell cycle and apoptosis of Epi-treated SW620 cells. Cells were transfected with 75 nM siC and siHuR (A, C, E), or with 2 μg/ml HA and HuR (B, D, F). After 24 h, cells were treated with Epi (5 μg/ml) for 24 h. Cells were then collected for the following analysis. **(A, B) The effect of HuR silencing and overexpression on cell cycle distribution of Epi-treated SW620 cells.** Cells were fixed and stained with PI then analyzed for cell cycle distribution by flow cytometry. The percentage of cells in each cell cycle stage was calculated and means from three independent experiments plotted. Statistical significance comparing the sub-G1 population was shown. ^#^ P<0.05 compared to the siC (A) or HA (B) group; ^Φ^ P<0.05 compared to the siHuR (A) or HuR (B) group; ^Δ^ P<0.05 compared to the siC + Epi (A) or HA + Epi (B) group. **(C, D) The effect of HuR knockdown and overexpression on apoptotic population of Epi-treated SW620 cells.** The apoptotic population of cells was detected by Annexin V apoptosis assay and analyzed by flow cytometry. The mean percentages of early apoptotic population (bottom right) and late apoptotic population (top right) from three independent experiments were calculated then the means ± S.D. were shown in each represented panel. **(E, F) The effect of HuR silencing and overexpression on the intrinsic and extrinsic apoptosis pathway of Epi-treated SW620 cells.** Cells were harvested for detection of the endogenous activity of caspase proteins by Caspase-Glo® 3/7, 8, 9 assay system and analyzed by Luminescence immunoassay analyzer. The relative percentage of caspase activity was calculated by setting the mean luminescence intensity of siC (E) or HA (F) group as 100%. Data are presented as means ± S.D. from three independent experiments. ^#^ P<0.05 compared to the siC (E) or HA (F) group; ^Φ^ P<0.05 compared to the siHuR (E) or HuR (F) group; ^Δ^ P<0.05 compared to the siC + Epi (E) or HA + Epi (F) group.

The cytotoxic effect of Epi is known to act through inducing mitochondrial apoptotic pathway [27,30]. The above finding led us to investigate whether HuR also regulated the Epi-induced apoptotic pathway. First, the effect of siHuR or overHuR on cell cycle distribution of SW620 cells treated with or without Epi was analyzed. HuR silencing increased the sub-G1 population, while HuR upregulation decreased it (Fig 5A and 5B: lane 2 vs lane 1; *P* < 0.05). Epi treatment alone also increased sub-G1 population as reported (Fig 5A and 5B: lane 3 vs lane 1; *P* < 0.05). Combining siHuR and Epi treatment further increased sub-G1 population compared to siHuR or Epi treatment alone (Fig 5A lane 4 vs lane 2 and 3, separately; *P* < 0.05). On the other hand, HuR overexpression protected the cells from the sub-G1 accumulating effect of Epi (Fig 5B lane 4 vs lane 3; *P* < 0.05). Therefore, HuR might regulate apoptosis pathway directly or affect Epi-induced apoptosis pathway. We next analyzed the effect of siHuR or overHuR on viable or death population of SW620 cells treated with or without Epi. Consistent with the above results, suppression of HuR escalated early apoptotic (Annexin V positive/PI negative) population, while upregulation of HuR diminished it. Epi treatment enhanced early and late apoptotic or necroptotic (Annexin V positive/PI positive) population. The combined treatment of siHuR and Epi further augmented late apoptotic or necroptotic population, while HuR overexpression protected cells from Epi-mediated apoptotic effect (Fig 5C and 5D). However, the treatment of Epi in the presence or absence of siHuR or overHuR did not show significant effect on the population of late apoptosis or necrosis (Annexin V negative/ PI positive) compared to siC or HA, respectively (Fig 5C and 5D). Whether the effect of HuR on Epi-induced apoptosis was via intrinsic or extrinsic pathway was assessed by examining the activity levels of caspase proteins. The result has displayed that knockdown HuR intensified the activity levels of caspase-8, -9 and -3/7 (Fig 5E), while overexpression of HuR decreased only the activity levels of caspase-8 and -3/7, which are the downstream initiator and executor of extrinsic apoptosis pathway (Fig 5F). However, overHuR did not affect intrinsic apoptosis pathway with or without the induction by Epi (Fig 5F). Epi treatment only induced caspase-9 and -3/7, which are the downstream initiator and executor of intrinsic apoptosis pathway, respectively (Fig 5E and 5F). When combining siHuR and Epi, both intrinsic and extrinsic apoptosis pathways were further activated (Fig 5E).

### Epi-induced apoptosis mediated by HuR silencing was confirmed by changes in MMP and apoptosis-related gene expressions

**Fig 6:**
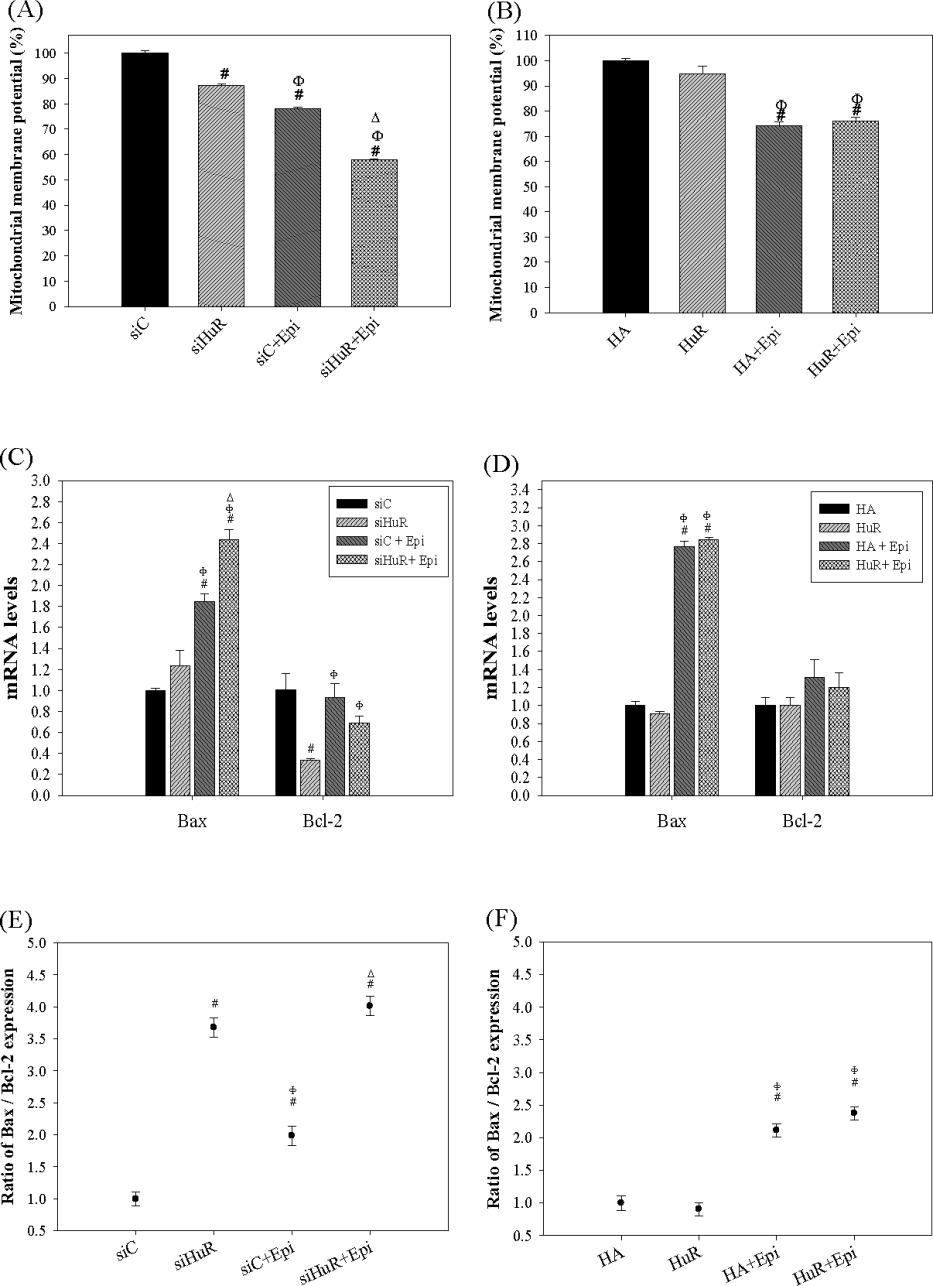
HuR regulated the Epi-induced intrinsic apoptosis pathway of SW620 cells. **(A-B) The effect of HuR silencing and overexpression on the mitochondria membrane potential of Epi-treated SW620 cells.** Cells were transfected with 75 nM siC or siHuR (A), or 2 μg/ml HA or HuR (B). After 24 h, cells were treated with Epi (5 μg/ml) for 24 h, then harvested and stained by DiOC6 for measuring mitochondria membrane potential. The percentage of mitochondria membrane potential intensity was calculated by setting the mean fluorescence intensity of siC (A) or HA (B) group as 100%. Data are presented as means ± S.D. from three independent experiments. ^#^ P<0.05 compared to the siC (A) or HA (B) group; ^Φ^ P<0.05 compared to the siHuR (A) or HuR (B) group; Δ P<0.05 compared to the siC + Epi group. **(C-D) The effect of HuR knockdown and overexpression on the apoptosis-related genes of Epi-treated SW620 cells.** RNA was extracted from cells as designed in (A) and (B) for measurement of mRNA expressions of apoptosis-related genes by qPCR assay. The relative expression mRNA level was calculated by setting siC (C) or HA (D) group as 1. Data are presented as means ± S.D. from three independent experiments. ^#^ P<0.05 compared to the siC (C) or HA (D) group; ^Φ^ P<0.05 compared to the siHuR (C) or HuR (D) group; ^Δ^ P<0.05 compared to the siC + Epi group (C). **(E-F) The effect of HuR silencing and overexpression on the ratio of Bax versus Bcl-2 mRNA levels (Bax/Bcl-2) in Epi-treated SW620 cells.** The Bax/Bcl-2 ratio was calculated from the qPCR data of (C) and (D) then plotted in (E) and (F), respectively. The relative mRNA level of Bax was divided by that of Bcl-2 in each group and means ± S.D. from three independent experiments plotted. ^#^ P<0.05 compared to the siC (E) or HA (F) group; ^Φ^ P<0.05 compared to the siHuR (E) or HuR (F) group; ^Δ^ P<0.05 compared to the siC + Epi (E) group.

Epi is known to generate ROS which trigger mitochondrial disruption and thereafter activated intrinsic apoptotic pathway [21,27]. In the present study, we have detected that siHuR and overHuR affected ROS production and Epi accumulation. Interestingly, intrinsic apoptotic pathway was further induced by knockdown but not via overexpression of HuR. Therefore, it was possible that Epi-induced mitochondria-related apoptosis pathway was regulated by HuR silencing. We also compared the MMP change upon Epi treatment among cells expressing siHuR and overHuR. As expected, Epi treatment decreased MMP to 77.99 ± 0.75% and siHuR reduced MMP to 87.35 ± 0.49% compared to the control group. Moreover, the treatment of siHuR+Epi decreased the MMP level to 57.85 ± 0.34%, which was much lower than the individual siHuR and Epi treatment did (Fig 6A; *P* < 0.05). On the other hand, HuR overexpression had no further effect on the Epi-reduced MMP (Fig 6B; *P* > 0.05). This is correlated to the present result from the caspase activity assay that overexpression of HuR could not protect cells from Epi-induced apoptosis. Epi has also been known to alter expressions of several apoptosis-related genes, including Bax, and Bcl-2 [17,21,27]. We next examined whether HuR was involved in Epi-modulated gene expressions of Bax and Bcl-2. Epi increased the mRNA level of Bax, one pro-apoptosis gene (Fig 6C and 6D). Knockdown of HuR escalated the mRNA level of Bax, but diminished that of Bcl-2. Combining siHuR and Epi further amplified the mRNA level of Bax (Fig 6C). However, overexpression of HuR had no additional effect on these genes in SW620 cells treated with or without Epi (Fig 6D). Additionally, combining siHuR and Epi further escalated the mRNA level of Bax and mildly reduced the Bcl-2 expression in HCT116 cells (data not shown). Subsequently, the apoptosis induction was positively correlated to the ratio of Bax/Bcl-2. The result showed that both siHuR and Epi alone both increased Bax/Bcl-2. Combining siHuR and Epi treatment had the highest Bax/Bcl-2 ratio, which manifested the strongest apoptosis-triggering action of Epi, as potentiated by siHuR (Fig 6E). Again, overexpression of HuR showed no further effect on Epi-induced Bax/Bcl-2 (Fig 6F). These results support the previous deduction that siHuR promoted Epi-induced apoptosis, but HuR overexpression could not rescue cells from Epi-induced apoptosis.

### HuR silencing diminished the protein expressions of survival, MDR, and anti-apoptosis signaling pathways

**Fig 7:**
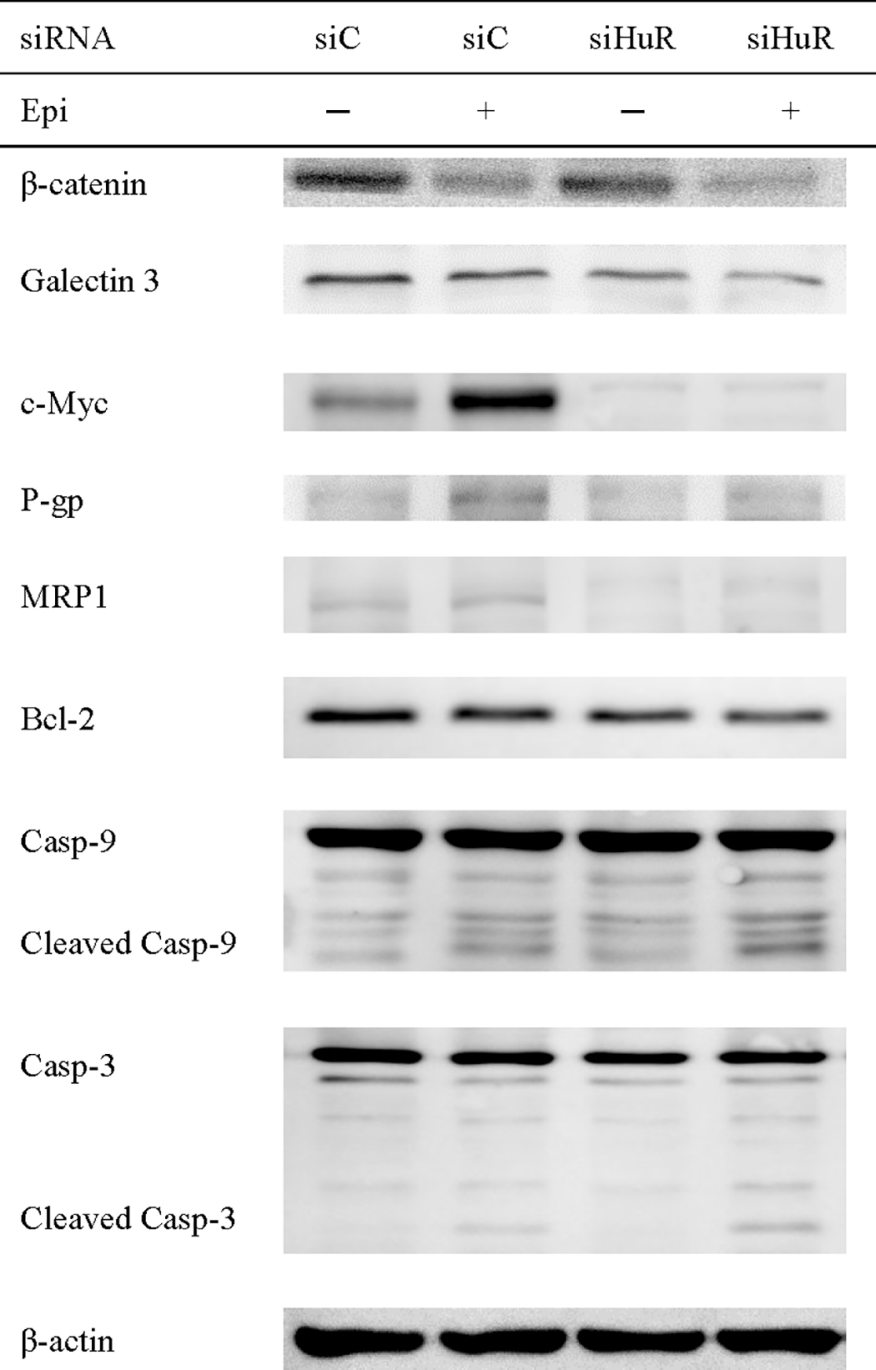
The effect of HuR silencing on the protein expression levels of survival signaling-, efflux transporter- and apoptosis-related factors in colon cancer cells by western blotting. Cells were transfected with 75 nM siC or siHuR. After 24 h, cells were treated with Epi (5 μg/ml) for 24 h and then harvested. The expression levels of proteins, including galectin-3, c-Myc, P-gp, MRP1, Bcl-2, caspase-3, -9 and β-actin were examined by western blotting (N = 3). The representative result is shown.

To confirm the pathways involved in the regulation caused by the treatments of siHuR and/or Epi, we evaluated the protein expressions of characteristic survival-, MDR-, and apoptosis-related factors by western blot. Consistently, HuR knockdown reduced the protein expressions of galectin-3, Bcl-2, P-gp and MRP1 compared with that of siC (Fig 7). Furthermore, caspase-3 and -9 was activated and cleaved after HuR silencing (Fig 7). Conversely, Epi treatment (Epi + siC) amplified the protein expressions of c-Myc, P-gp and MRP1. Interestingly, the co-treatment of Epi + siHuR diminished the expressions of galectin-3, ß-catenin, c-Myc, P-gp and MRP1, but further escalated the expressions of cleaved caspase-3 and -9 compared with that of Epi +siC, respectively (Fig 7).

## Discussion

Epi is a chemotherapeutic agent for the treatment of breast, gastric, colorectal, and ovarian cancers [31]. Although it is not the drug of choice for colon cancer therapy, Epi is used in this study as a model anticancer drug because it is a substrate of P-gp, MRP1, and MRP2. In our previous studies, Epi exhibits a potent apoptosis-inducing effect against diverse cancer cells, including human squamous cell carcinoma, pluripotent testicular embryonic carcinoma, colorectal carcinoma, and cervical cancer cells by way of the intrinsic mitochondrial signaling [17,22,30,32] via intracellular ROS production [21,22].

MDR is regulated by multiple signaling pathways. Among them, β-catenin is critical in regulating Wnt signaling pathway by entering the nucleus and binding to T-cell factor/lymphoid enhancer factor (TCF/LEF) and activating their specific target genes including c-Myc, cyclin D1, MDR1 in colon cancer and chronic myeloid leukemia (CML) cells [18,33,34]. HuR played an important role in modifying gene expressions of cancer-related mRNAs encoding numerous protooncogenes, cell cycle regulators, cytokines, chemokines, growth factors, invasion factors, and proinflammatory factors [2,3]. For example, c-Myc translation was regulated by cytoplasmic HuR binding to the 3'-UTR of c-Myc mRNA [35]. Moreover, cyclin D1 mRNA showed a high binding to HuR, indicating a positive correlation between HuR expression and cyclin D1 mRNA stability in renal mesangial cells [25]. In our previous study, we showed for the first time that the treatment of siRNA against galectin-3 sensitized MDR cells to Epi by inhibiting P-gp and MRPs and the activation of the mitochondrial apoptosis pathway through modulation of the β-catenin/GSK-3β pathway in human colon cancer Caco-2 cells [17]. In this study, we further verified that siHuR or overHuR modulated the change of chemoresistance of colon cancer cells to Epi through galectin-3/β-catenin signaling.

Our results supported that siHuR increased ROS generation (Fig 3), increased the expressions of GSK-3β (Fig 4C), reduced the expressions of galectin-3/β-catenin pathway (Figs 4C and 7) and the related oncogene cyclin D1 (Fig 4E). We further found that HuR silencing decreased MDR transporter expressions (Figs 4G and 7), enhanced Epi cellular accumulation (Fig 4A) and induced apoptosis pathway (Figs 5A, 5C, 5E, 6A, 6C, 6E and 7), leading to the increased cytotoxic effect of Epi on colon cancer cells (Fig 2B). In contrast, HuR overexpression protected SW620 cells from Epi-induced cytotoxicity (Figs 2C, 4B and 4H), mainly via modulation of apoptosis pathway (Figs 5B, 5D, 5F, 6B, 6D and 6F). Other growth-related signaling pathways were also possibly involved in HuR-mediated survival of cancer cells after Epi treatment (Fig 4D and 4F). Recent studies have proved that HuR controlled the galectin-3/β-catenin pathway to affect cell growth and apoptosis [4,14].

In this study, we confirmed that HuR affected cytotoxic effect of Epi via the galectin-3/β-catenin pathway, as shown in Figs 4 and 7. HuR knockdown diminished the expressions of ABC transporters, thus enhancing the uptake of Epi into cancer cells to intensify chemosensitivity of SW620 cells to Epi (Figs 2B, 4G and 7). Two regulator genes, cyclin D1 and c-Myc, are downstream oncogenes of galectin-3/β-catenin pathway. In the present study, we also investigated the expressions of the Wnt signaling-related genes cyclin D1 and c-Myc upon HuR knockdown or upregulation and/or Epi treatment. The result demonstrated that HuR silencing decreased the cyclin D1 expression (Fig 4E), which was correlated to the enhancing effect of siHuR on the cytotoxicity of Epi (Fig 2B). Consistently, suberoylanilide hydroxamic reduced mouse epidermal Cl41 cell proliferation through inhibiting the mRNA stability and protein expression of cyclin D1, as mediated by the decrease in HuR expression [13]. A ribozyme against HuR decreased HuR expressions and inhibited breast cancer cell growth and invasion with a concomitant reduction in the levels of cyclin D1 [36]. Interestingly, mRNA level of c-Myc was negatively regulated by HuR (Fig 4E and 4F), but siHuR decreased the protein expression of c-Myc (Fig 7). It has been reported that c-Myc could trigger death receptor-induced apoptosis pathway [37]. In addition, overexpression of c-Myc caused improved sensitivity of pancreatic cancer cells to cisplatin-induced apoptosis [38]. We found that siHuR might also induce extrinsic apoptotic pathway, as evidenced by our result of caspase 8 activity assay (Fig 5F). However, the post-transcriptional regulation of c-Myc by siHuR might reduce the protein expression of c-Myc (Fig 7), thus inhibiting the role of c-Myc in augmenting Epi-mediated MDR transporter-related resistance, as shown by the reduced protein expressions of P-gp and MRP1 (Fig 7). We thus suggested that siHuR might intensify the sensitization of colon cancer cells to Epi, via triggering apoptosis and suppressing ABC transporters (Figs 4–7).

**Fig 8:**
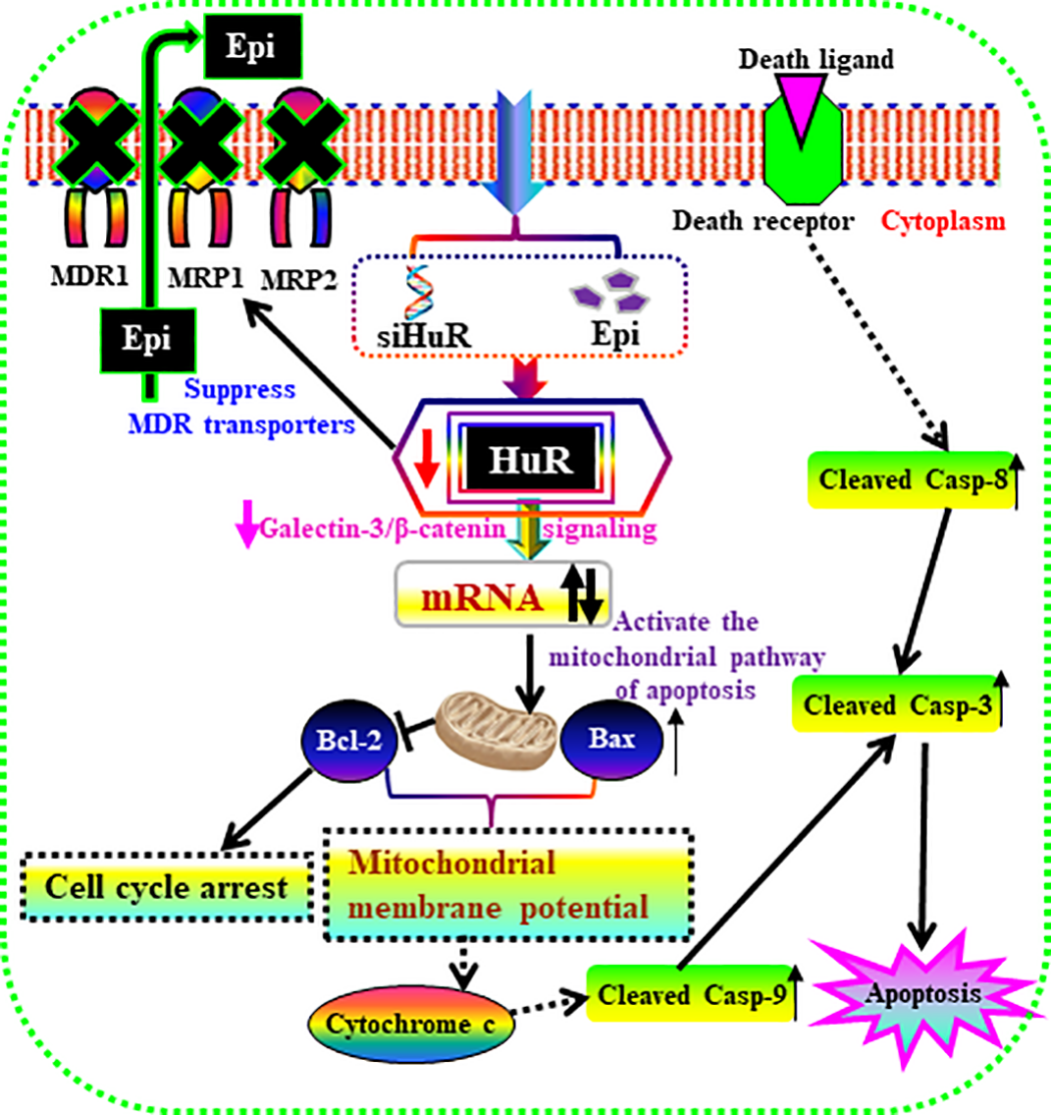
The multiple signaling pathways modulated by siHuR for inhibiting galectin-3/β-catenin signaling, suppressing ABC transporters and inducing apoptosis to reverse Epi-mediated MDR in colon cancer cells.

Consistent with our results, ellagic acid (EA) remarkably enhanced ROS levels, down-regulated HuR, and diminished intracellular levels of β-catenin, thus activating apoptosis via upregulation of caspase-3 and p21 in the human prostate cancer LNCaP cells [39]. Furthermore, HuR silencing increased the Bax/Bcl-2 ratio and caspase-3 activity, supporting the inhibition in carcinogenesis and tumor suppression [40]. Additionally, the long intergenic noncoding RNA UFC1 interacted directly with HuR to increase β-catenin expression, leading to proliferation enhancement and apoptosis suppression in hepatocellular carcinoma cells (HCC) cells, and intensified growth of xenograft tumors in mice [14]. Collectively, we sum up that HuR may control galectin-3/β-catenin signaling pathway and coordinately regulate sequential transcriptional expressions of galectin-3, β-catenin, cyclin D1, Bcl-2, c-Myc, P-gp, and MRP, thus leading to the reversal of Epi-resistance mediated by ABC transporters and anti-apoptotic pathways. The multiple signaling pathways modulated by siHuR for inhibiting β-catenin signaling, suppressing ABC transporters and inducing apoptosis to reverse Epi-mediated MDR in colon cancer cells are shown in Fig 8.

## Conclusions

Collectively, inhibition of galectin-3/β-catenin signaling pathway via post-transcriptional control by siHuR may provide a powerful regimen for reversing MDR in colon cancer. To our best knowledge, this is an innovative investigation linking HuR silencing to oncogene regulation, efflux transporter reversal and apoptosis modulation for improving chemosensitivity of colon cancer cells to antineoplastic agents.
